# Charge Accumulation in the Homo-Crosslinked-Polyethylene Bilayer

**DOI:** 10.3390/ma15093024

**Published:** 2022-04-21

**Authors:** Wei Zhao, Huaqiang Li, Wenpeng Li, Xin Chen, Lisheng Zhong, Jinghui Gao

**Affiliations:** 1 State Key Laboratory of Electrical Insulation and Power Equipment, Xi’an Jiaotong University, Xi’an 710049, China; verazhaowei0513@163.com (W.Z.); lhqxjtu@mail.xjtu.edu.cn (H.L.); 2 State Grid Smart Grid Research Institute Co., Ltd., Beijing 102202, China; lwp1017@126.com (W.L.); chenxin1@geiri.sgcc.com.cn (X.C.)

**Keywords:** homo-crosslinked-polyethylene bilayer, charge accumulation, homo-junction region, factory joint return insulation

## Abstract

The homo-crosslinked-polyethylene (H-XLPE) bilayer simplifies the returned insulation structure of the factory joint in submarine cables, and its dielectric property is key to the reliability of the power transmission system. In this paper, we investigated the charge accumulation phenomenon in a secondary thermocompression H-XLPE bilayer using the pulse electro-acoustic method. The charge accumulation reduces its overall breakdown strength when compared with XLPE. According to X-ray diffraction measurement and thermal analysis results, the specimen forms a homo-junction region between the bilayers, which has overlapping spherulites with a thick lamella, high crystallinity, and high surface free energy. The charge accumulation can be ascribed to fused lamellas and the crystal imperfection of the homo-junction region, which restricts the charge transport process and exhibits a higher number of deep traps. This study emphasizes the importance of the homo-junction region in the H-XLPE bilayer, which should be considered in the design and operation of factory joint insulation.

## 1. Introduction

Due to its excellent dielectric properties and mechanical and chemical stability, crosslinked polyethylene (XLPE) insulation material is widely used in power cables [1,2,3,4], such as in submarine cables and factory joints, usually connecting two cables. The factory joint uses XLPE return insulation, which is formed on the cable insulation by the extrusion and vulcanization of the same insulating material. Such a complex insulation structure, which is one of the most important issues related to cable operation safety, can be simplified by the use of a XLPE bilayer [5,6]. 

Tremendous efforts have been made to investigate the dielectric properties of XLPE bilayer to meet the reliability requirements of submarine cable systems. On the one hand, it has been found that the bilayer structure exhibits significant diversity in alternating current (AC) breakdown strength due to the variations in crosslinking degree and byproduct content [7,8]. On the other hand, it has been discovered that interfacial microcracks can preferentially initiate an electric tree that rapidly grows to trigger partial discharge and as a result, insulation is damaged [9,10]. The micropore located on the interface can improve current density and break elongation of the material [11]. This brief survey indicates that most of the works have concentrated on the diversity of bilayer structure and the interface effect, the so-called extrinsic effect. However, studies on the intrinsic characteristic of the XLPE bilayer insulation structure are still missing. The pulse electro-acoustic (PEA) analysis has proven to be an effective means of charge characterization in dielectric materials [12,13,14,15]. 

In this paper, the homo-XLPE (H-XLPE) bilayer and its intrinsic characteristics are investigated. In order to construct the intrinsic structure of the XLPE bilayer, each layer follows a consistent approach, and the material variations for layers is eliminated with relatively smooth interface in between. The charge accumulation in H-XLPE bilayer and its effect on direct current (DC) breakdown strength are studied. Then to explain this phenomenon, the crystal morphology in the H-XLPE bilayer is characterized, which includes spherulite surface morphology, microcrystal size, crystallinity, and surface free energy. Finally, the charge accumulation is discussed considering the formation of the homo-junction region. This paper highlights a key issue in the H-XLPE bilayer and provides a solid foundation for factory joint insulation research.

## 2. Materials and Methods

### 2.1. Material Preparation

Low-density polyethylene (LDPE) with a density of 0.922 g/cm^3^ and melting index of 2 g/10 min (190 °C, 2.16 kg) was selected as the raw material, which was dried at 80 °C for 12 h before specimen preparation. LDPE granules were melted at 115 °C, 10 MPa for 5 min; afterward, they were crosslinked at 180 °C, 15 MPa for 10 min, and then cooled with circulating water at 15 MPa for 15 min in the vulcanizer. XLPE plate specimens with a thickness of 100 μm and 200 μm were prepared. These specimens had a surface roughness of about R_a_ = 100 nm. The specimens with a thickness of 5 mm and 10 mm were obtained by increasing the melting time and crosslinking time to 30 min and 20 min, respectively. Then, we superimposed two pieces of 100 μm or 5 mm XLPE plate specimen together and hot pressed again for 20 min at 180 °C and 15 MPa. In this way, H-XLPE bilayer specimens with a thickness of 200 μm and 10 mm were prepared. 

**Figure 1:**
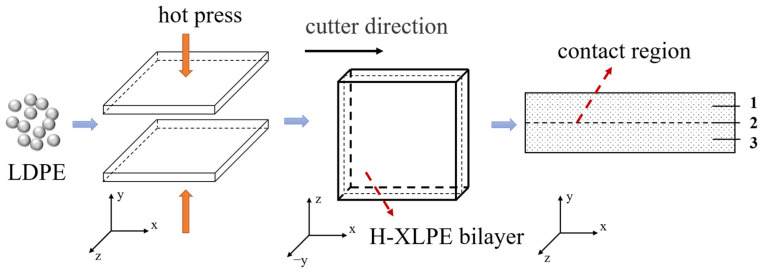
Schematic diagram showing the sequence of specimen preparation.

The specimens with a thickness of 200 μm were used for charge distribution and DC breakdown measurement. The slice used for crystal morphology characterization was cut from a specimen with a thickness of 10 mm using a precision control machine. The surface of the sliced specimen was polished with 2000 mesh sandpaper to ensure smoothness, as required for the optical measurement. The sliced specimen was divided into three equal parts to study the crystal morphology, labeled as 1, 2, 3 in H-XLPE bilayer and 1’, 2’, 3’ in XLPE, with position 2 representing the contact region between the bilayers. All the prepared specimens were placed in an oven at 80 °C for 12 h to volatilize the impurities in the substance. The specimen preparation sequence is shown in Figure 1.

### 2.2. Charge and DC Breakdown Measurement

The charge distribution in a specimen is measured using the PEA, in which the cathode is made of semi-conductive material and the anode is made of aluminum. The electric field strength is calibrated at 10 kV/mm and measured at 30 kV/mm for 120 min; then, the electric field is removed, and the decay charge is measured for another 120 min. 

The DC breakdown measurement makes use of a cylindrical metal electrode with a diameter of 25 mm and a radius of chamfer equal to 2 mm. The electrode system and specimen were immersed in insulating oil to avoid flashover at an ambient temperature of 30 °C. Then, a high voltage was applied at a ramp rate of 1 kV/s until the breakdown occurred. The data of 12 effective breakdowns was analyzed by Weibull distribution, with a confidence interval of 95%.

### 2.3. Crystal Morphology Characterization

The slice specimen was immersed in H_2_SO_4_ and KMnO_4_ mixed corrosive solution for 4 h at 30 °C. After etching, the specimen was washed with diluted sulfuric acid, hydrogen peroxide, deionized water, and acetone before being dried in a fume hood. After gold sputtering, the spherulite surface morphology was analyzed by a scanning electron microscope (SEM, Keyence, Osaka, Japan).

X-ray diffraction (XRD, Bruker, Karlsruhe, Germany) was used to determine microcrystal size of different lattice plane orientations. For this measurement, 40 kV potential and 40 mA current were applied to the specimen, and the scanning angle was varied from 10° to 40° at a scanning rate of 2°/min at 30 °C. Jade 6 software was used to analyze XRD data, which can realize peak fitting. Small Angle X-ray Scattering (SAXS, Bruker, Karlsruhe, Germany) was employed to analyze the periodic interval length between crystalline phases. The X-ray wavelength was 0.154 nm and the scanning angle was from 0.1° to 2.3°.

The crystallinity and the surface free energy were measured with the help of a differential scanning calorimeter (DSC, Mettler Toledo, Zurich, Switzerland). The specimen mass was 8 ± 0.5 mg, and the test temperature ranged from 50 °C to 150 °C at a heating rate of 10 °C/min in nitrogen.

## 3. Results

### 3.1. Charge Characteristics

**Figure 2:**
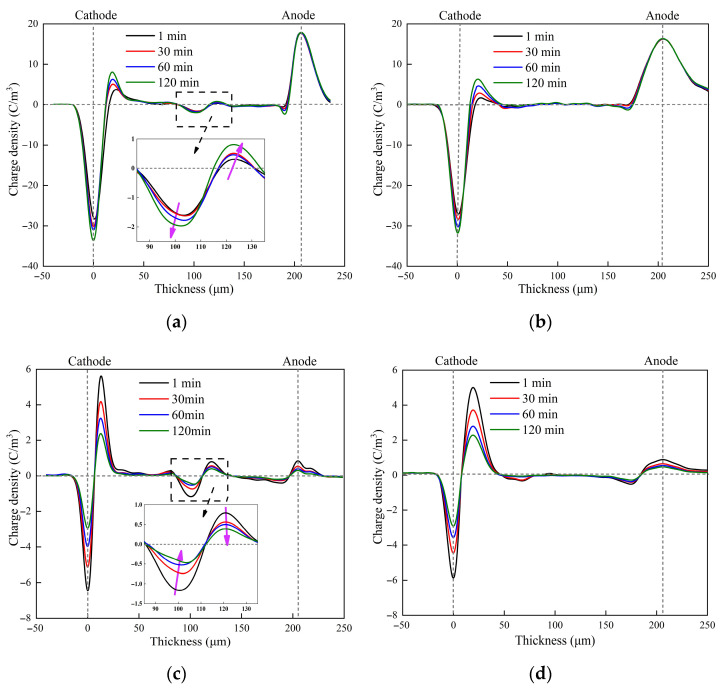
The charge distribution: (**a**) polarization in H-XLPE bilayer; (**b**) polarization in XLPE; (**c**) depolarization in H-XLPE bilayer; (**d**) depolarization in XLPE.

The charge distribution under a polarization electric field of 30 kV/mm applied for 120 min to H-XLPE bilayer and XLPE is shown in Figure 2a,b, respectively. The accumulation of positive charge near the cathode and negative charge near the anode can be seen in the two specimens, and the heteropolar charge increases with the polarization time. Significant charge accumulation occurs in the contact region of the H-XLPE bilayer (see Figure 2a), which does not occur in XLPE, resulting in a flat charge distribution curve. The initial charge density is 1.6 C/m^3^ as the voltage is applied, but the charge begins to accumulate and eventually reaches 2 C/m^3^ as the polarization time increases. The residue charge distribution in H-XLPE bilayer and XLPE during depolarization is shown in Figure 2c,d, respectively. The accumulated charge in the H-XLPE bilayer dissipates quickly: the negative charge is reduced to about 0.5 C/m^3^ and the positive charge is reduced to about 0.4 C/m^3^ after depolarization of 120 min. 

### 3.2. Electric Field Distribution and Breakdown Characteristics

**Figure 3:**
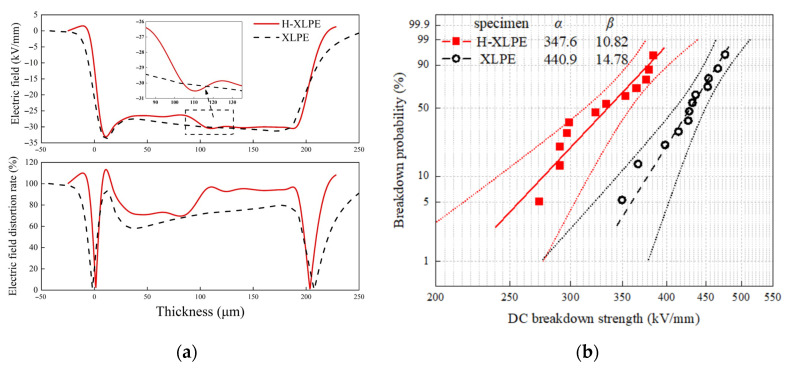
Effect of charge accumulation: (**a**) electric field distribution (upper panel) and distortion rate (lower panel); (**b**) Weibull distribution of DC breakdown strength.

The effect of charge accumulation on electric field distribution and breakdown characteristics also needs to be further discussed, considering its application in factory joint return insulation of the submarine cable. The electric field *E_C_*(*x*) produced by charge accumulation can be calculated according to Equation (1) [12,16,17]:

(1) $$\frac{dE_{c}}{dx}+\frac{1}{x}E_{a}=\frac{\rho (x)}{\varepsilon_{0}\varepsilon_{r}}$$

where *E_a_* is the applied electric field, *ρ*(*x*) is charge density, *ε*_0_ is the vacuum permittivity (8.85 × 10^−12^ F/m), and *ε_r_* is the relative permittivity (2.2). The electric field distribution in H-XLPE bilayer and XLPE under polarization for 120 min is shown in the upper panel of Figure 3a. The electric field of the H-XLPE bilayer is lower in the thickness range of 40–105 μm and 115–190 μm, and higher in the thickness range of 105–115 μm, when compared to the XLPE specimen. The reason is that the electric field formed by the accumulated charge and the heteropolar charge near the electrode due to opposite field direction weakening the applied electric field strength. The accumulated charge, on the other hand, generates a local electric field that then strengthens the electric field strength in the thickness range of 105–115 μm. The uniform distribution caused by charge accumulation can be characterized by the electric field distortion rate as in Equation (2) [18]:

(2) $$\delta (x)=\left|\frac{d\cdot E(x)}{\int_{0}^{d}\left|E(x)\right|dx}-1\right|\times 100\%$$

where *δ*(*x*) is the electric field distortion rate at the position with distance *x* from the cathode, and *d* is the specimen thickness. *E*(*x*) describes spatial variation of the electric field. The electric field distortion rate is exhibited in the lower panel of Figure 3a. The maximum electric field distortion rate occurs at the cathode, which is caused by a heteropolar charge in both specimens. However, the electric field distortion rate in the H-XLPE bilayer is higher than that in XLPE, especially near a thickness of 110 μm. 

The breakdown strength can be calculated by the two-parameter Weibull formula as in Equation (3):

(3) $$F(y)=1-\exp(-{\left(\frac{y}{\alpha }\right)}^{\beta })$$

where *y* is the breakdown strength, *F*(*y*) is the failure probability, *α* is the scale parameter that represents the breakdown strength when the failure probability is 63.2%, and *β* is a shape parameter that accounts for the data dispersion. Figure 3b shows the Weibull distribution of DC breakdown strength of H-XLPE bilayer and XLPE at 30 °C. The breakdown strength of the H-XLPE bilayer is 21.16% lower than that of XLPE, and the breakdown data of the H-XLPE bilayer is more dispersed. The electric field distortion caused by charge accumulation most likely degrades the dielectric strength of the H-XLPE bilayer. Structure analysis is required to understand this phenomenon.

### 3.3. Trap Characteristics

**Figure 4:**
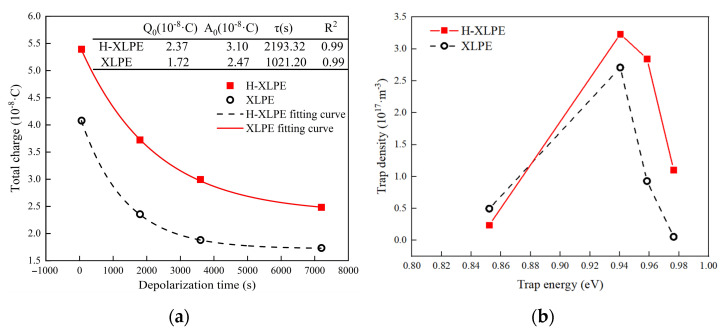
Trap characteristics: (**a**) the charge attenuation versus depolarization time; (**b**) trap density versus trap energy.

The dissipation of accumulated charge can reflect the trap characteristics in polymer material during the depolarization process, implying that the charge accumulates in the shallow trap first and then collapses in the deeper trap [19]. Based on the residue charge distribution shown in Figure 2c,d, the attenuation of total charge *Q*(*t*) can be obtained by integrating the charge density curve as in Equation (4):

(4) $$Q(t)=\int_{0}^{d}\left|\rho (x,t)\right|\cdot Sdx$$

where *ρ* is the charge linear density, and *S* is the contact area of the electrode and specimen. The charge attenuation in 50–150 μm thickness is shown in Figure 4a. The charge decreases rapidly at the start of the depolarization, and the attenuation tends to saturate after about 3600 s. In the depolarization process, the total charge and residual charge in the H-XLPE bilayer are greater than those in XLPE, indicating that more traps, especially deep traps, exist in the H-XLPE bilayer. The data are fitted with an exponential function:

(5) $$Q(t)=Q_{0}+A\exp(-\frac{t}{\tau })$$

where *Q*_0_ is the residual charge, *A* is the coefficient, and *τ* is the decay time constant. The fitting parameters are listed in Figure 4a. The fitting coefficient (*R*^2^) of the two specimens is 0.99, which means that the charge attenuation follows the exponential attenuation law quite well. 

Assuming that the charge would not be trapped again after collapsing, the trap energy *E_t_* and trap density *N_t_*(*E_t_*) can be calculated by using Equations (6) and (7), respectively [20].

(6) $$E_{t}=kT\cdot \ln\nu t$$

(7) $$N_{t}\left(E_{t}\right)=\frac{2A_{0}r^{\prime }t}{d^{2}q_{0}kT\tau f_{0}\left(E_{t}\right)}\cdot \exp\left(-\frac{t}{\tau }\right)$$

where *k* is the Boltzmann constant (8.568 × 10^−5^ eV), *T* is the absolute temperature, *ν* is the electron vibration frequency (3 × 10^12^ Hz), *r*’ is the average charge center of gravity (120 pm), *q*_0_ is the electron charge (1.6 × 10^−19^ C), and *f*_0_(*E_t_*) is the initial trap occupancy (0.5). The trap density versus trap energy for 50–150 μm thickness of both specimens is shown in Figure 4b. Both specimens achieved maximum trap density at a trap energy of about 0.94 eV. However, when the trap energy exceeds 0.94 eV, the trap density in H-XLPE bilayer is significantly higher than that in XLPE, where the maximum difference of trap densities of the two samples is about 1.95 × 10^17^ m^−3^ at 0.96 eV. The trap leading to charge accumulation requires further discussion from a crystal morphology perspective.

### 3.4. Spherulite Surface Morphology

**Figure 5:**
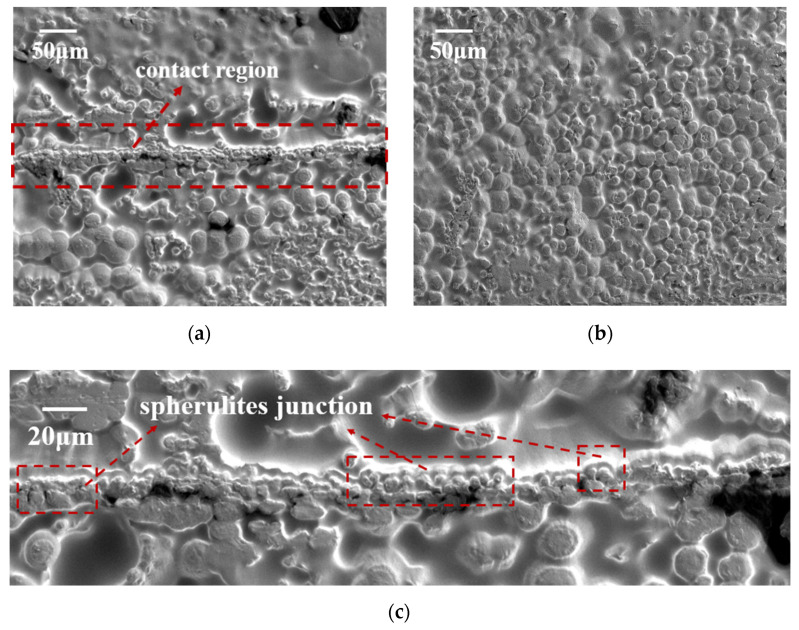
The spherulite surface morphology: (**a**) H-XLPE bilayer; (**b**) XLPE; (**c**) enlarged image of contact region of H-XLPE bilayer.

To understand the charge accumulation, the crystal morphology in the H-XLPE bilayer is further analyzed. As shown in Figure 5a,b, the surface morphology of spherulites can reflect the grain distribution and the cross sections of H-XLPE bilayer and XLPE specimens after corrosion. The spherulite diameters of the two specimens are in the range of 10–20 μm, but this statistic excludes the charge accumulation region of the H-XLPE bilayer, which is shown in Figure 5c. The spherulites with a diameter less than 10 μm no longer present original surface morphology; instead, they are the result of partially overlapping of the spherulites, which displays a compact spherulite junction (see dashed boxes in Figure 5c). This formation of the spherulite junction region may be an important reason for the increase in charge trap density.

### 3.5. Microcrystal Size and Periodic Crystal Morphology

**Figure 6:**
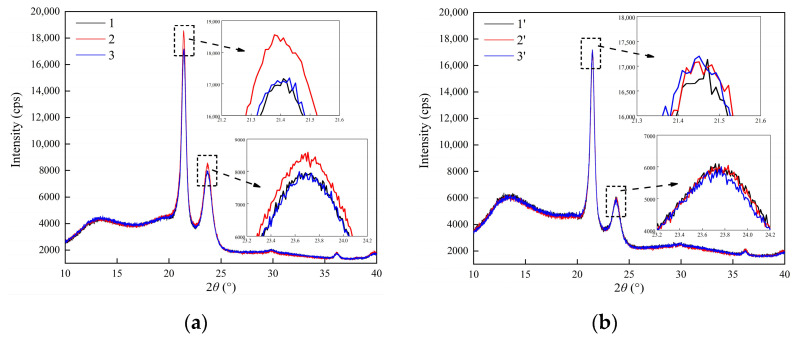
Lattice plane diffraction curve of three specimen positions: (**a**) H-XLPE bilayer; (**b**) XLPE.

The internal spherulite morphology—including the microcrystal size, the proportion of transition region, and amorphous region—is related to charge transport and thus requires further investigation. The lattice plane diffraction curves of the two specimens are shown in Figure 6. The spectra show two main lattice plane orientations, (110) and (200), which correspond to the diffraction peaks near 21.4° and 23.9°, respectively. The diffraction peak of position 2 is 10% higher than that of the other positions in the H-XLPE bilayer, but the curves of positions 1’, 2’, and 3’ in XLPE coincide, indicating that the lattice plane distribution is uniform in this case. This suggests that the lamella in the contact region is varying.

The microcrystal size of different lattice plane orientations can be analyzed by fitting the diffraction curve and applying Scherrer’s Formula:

(8) $$L=\frac{K\lambda }{B\cos\theta }$$

where *L* is the microcrystal size, *K* is the Scherrer constant (0.89), *λ* is the wavelength (0.15418 nm), *B* is the half peak width, and *θ* is the diffraction angle (°). *B* and *θ* can be obtained by the fitting result when the residual error is less than 5% in Jade 6. The estimated microcrystal size of both specimens at three different positions is shown in Table 1. No significant difference in the values of microcrystal size at three positions of the XLPE specimen can be seen. Furthermore, the microcrystal size of (110) and (200) lattice plane orientation at position 2 are 17.37 nm and 8.19 nm, respectively. The microcrystal size is slightly higher than that in the other two positions of the H-XLPE bilayer and also higher than that of all three positions of the XLPE specimen. The macromolecular chains in the contact region of the H-XLPE bilayer form thick lamella.

**Figure 7:**
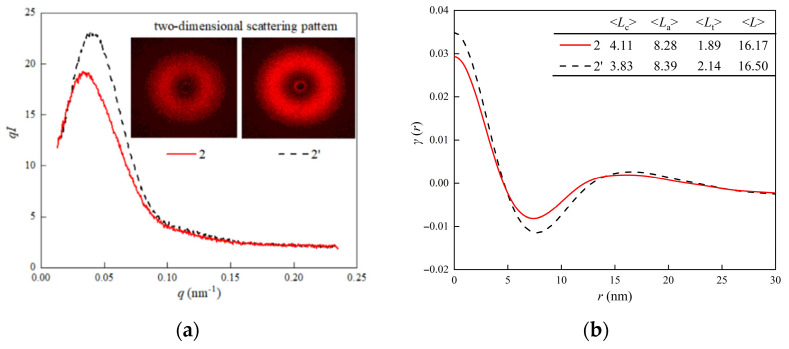
Small-angle scattering spectrum: (**a**) *I*(*q*)-*q* curves, where the inset gives two-dimensional scattering patterns of both specimens; (**b**) one-dimensional correlation function curve, where the table gives the periodic crystal morphology parameters.

Based on the lattice plane diffraction result, the periodic crystal morphology of thick lamella in the contact region should be considered. As shown in Figure 7a, the two-dimensional scattering rings of both specimens are symmetric, which indicates that their crystal morphology is isotropic. The scattering intensity curve *I*(*q*) and scattering vector *q* are obtained by the integral of the two-dimensional scattering pattern. The scattering peak intensity of position 2 of the H-XLPE bilayer is lower than that of XLPE, which indicates that the electron density in the contact region is lower. To obtain the periodic crystal morphology parameter in lamellas stacking direction, the one-dimensional correlation function *γ*(*r*) can be calculated by the inverse Fourier transform of *I* [21,22] using Equations (9) and (10):

*q* = (4*π*/*λ*) sin*θ* (9)

(10) $$\gamma (r)=\frac{\int_{0}^{\infty }q^{2}I(q)\cos qrdq}{\int_{0}^{\infty }q^{2}I(q)dq}$$

where *r* is spatial position. 

The one-dimensional correlation function curves of the two specimens are shown in Figure 7b. There is a transition phase between the crystalline phase and the amorphous phase because the linear part of the one-dimensional correlation curve does not start at zero; thus, the statistical thickness of the three-phase system (crystalline phase–transition phase–amorphous phase) is the focus of periodic crystal morphology. According to the literature [23], the horizontal ordinates of the start and end of the linear correspond to the thickness of transition region <*L*_t_> and crystalline region <*L*_c_>, respectively. The second peak position is periodic interval length between crystalline regions <*L*>. The thickness of the amorphous region <*L*_a_> can be calculated by using Equation (11); the periodic crystal morphology parameters are listed in the table in Figure 7b. The crystalline region in the contact region of the H-XLPE bilayer is thicker and accounts for more in a longer period as compared with XLPE. The proportion of (110) lattice plane orientation in XLPE is smaller than that in the contact region of the H-XLPE bilayer; therefore, it can be inferred that the thick lamella occurs in the (110) lattice plane orientation. The decrease in the transition region and the length of the periodic interval indicate that the thick lamella is more likely to be formed by the fusion of two adjacent lamellas and the coverage of the transition region. The charge transportation process is hampered by thicker lamella, smaller transition, and amorphous regions.

<*L*_a_> = <*L*> − <*L*_c_> − 2<*L*_t_> (11)

### 3.6. Crystallinity and Surface Free Energy

**Figure 8:**
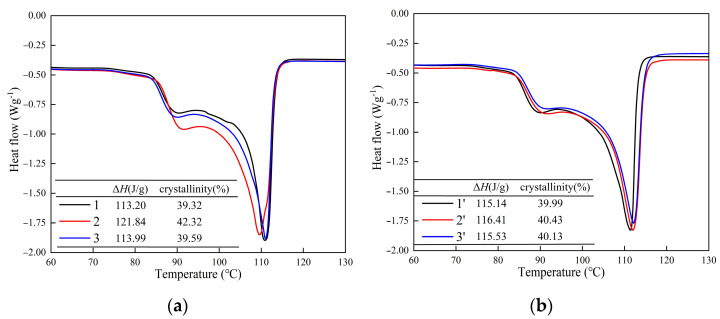
Melting curves, (**a**) H-XLPE bilayer; (**b**) XLPE.

Crystal properties such as crystallinity and surface free energy can further explain the long period and represent the spherulite integrity, so it is necessary to pay attention to the contact region of the H-XLPE bilayer. The specimens’ melt curves based on the following equation are shown in Figure 8.

(12) $$X_{C}=\frac{\Delta H}{\Delta H_{100}}\times 100\%$$

where *X_C_* is the crystallinity, Δ*H* is the melting enthalpy, and Δ*H*_100_ is the melting enthalpy when the specimen reaches 100% crystallization (287.9 J/g). The calculated crystallinities at various positions of both specimens are listed in Figure 8. The crystallinity of position 2 in the H-XLPE bilayer is greater than that of other positions, manifesting that the contact region contains more macroscopically arranged macromolecular chains. The melting peak in the range of 85–90 °C, which represents little spherulite crystallization, can be seen at position 2 in the H-XLPE bilayer greater than at other positions in two specimens. This observation is in line with the spherulite surface morphologies shown in Figure 5c.

The melting temperature in position 2 is smaller, which is related to the spherulite’s surface free energy. According to Gibbs’ Formula,

(13) $$T_{m}=T_{m0}\cdot \left(1-\frac{2\sigma_{e}}{\Delta H_{m0}L}\right)$$

where *T*_m_ is the melting temperature, *T*_m0_ is the melting temperature of the infinite lamella (414.6 K), *σ*_e_ is the surface free energy, and *H*_m0_ is the melting enthalpy of unit volume (2.88 × 10^8^ J/m^3^). As shown in Table 2, the surface free energy in position 2 of the H-XLPE bilayer is higher than that of the other positions. The surface free energy is usually related to the spherulite integrity [24,25], meaning that a spherulite with lower surface free energy exhibits better integrity, and vice versa. The spherulite integrity in the contact region of the H-XLPE bilayer is changed, and there may be more incomplete spherulites or defects that are more attractive to charge, resulting in charge trapping.

## 4. Discussion

**Figure 9:**
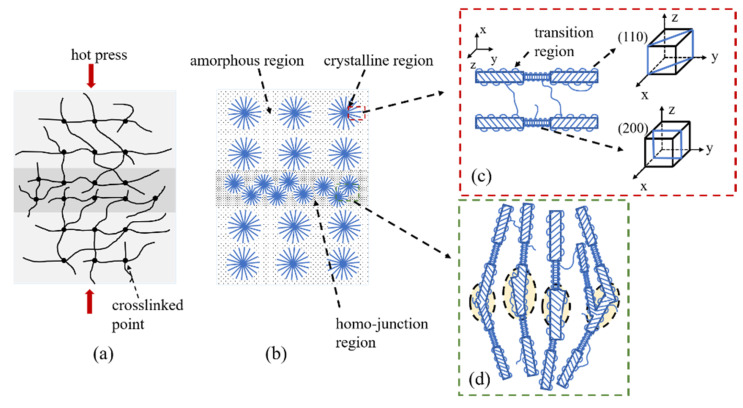
Schematic diagram of the H-XLPE bilayer: (**a**) crosslinked network during melting; (**b**) spherulites during cooling; (**c**) lattice plane orientation; (**d**) homo-junction region.

As the charge transport process during polarization is related to the polymer structure, the charge accumulation in H-XLPE bilayer is discussed from the perspective of crystal morphology. The adjacent spherulites in H-XLPE bilayer are overlapping (Figure 5c), and as compared to the average value of XLPE specimen, the corresponding crystal morphology in the contact region of H-XLPE bilayer possesses larger microcrystal size ((110): 17.37 nm > 16.41 nm, (200): 8.19 nm > 7.67 nm), less amorphous and transition regions in the periodic interval (<*L*_t_>: 1.89 nm < 2.14 nm, <*L*_a_>: 8.28 nm < 8.39 nm), higher crystallinity (42.32% > 40.18%), and higher surface free energy ((110): 193.77 × 10^−3^ J/m^2^ > 147.92×10^−3^ J/m^2^, (200): 91.31 × 10^−3^ J/m^2^ > 69.14 × 10^−3^ J/m^2^). As a result, a homo-junction region is created, which is explained in Figure 9.

The crosslinked network in H-XLPE bilayer exists before the formation of the crystalline structure because the crosslinked reaction occurrs during the process of heating up to 180 °C, whereas the formation of the crystalline structure takes place during the cooling process. As the temperature drops, the macromolecular chains gradually freeze and fold into a lattice, with the crosslinked point as a crystal nucleus [26,27,28]; then, lamella is created and finally stacks into spherulite. The spherulite diameter reduces, and even the adjacent spherulites that are prone to partial overlapping are restricted by the growth space because the distance between crosslinked points in the contact region of the H-XLPE bilayer becomes short under hot pressing. Meanwhile, the neighboring lattices are no longer independent, and the macromolecular chains will be arranged into the fused lattices to form thick lamella with high crystallinity. Furthermore, in such fused lamellas, the ends of macromolecular chains and vacancies are more likely to appear, resulting in a decrease in spherulite integrity and an increase in surface free energy. Consequently, the overlapping spherulites with a thick lamella, high crystallinity, and high surface free energy can be considered as a homo-junction region. According to different folding directions of macromolecular chains, XLPE lamella mainly exhibits (110) and (200) lattice plane orientation, and lamellas fusion tends to occur on the (110) lattice plane (Table 1 and Figure 7b). 

**Figure 10:**
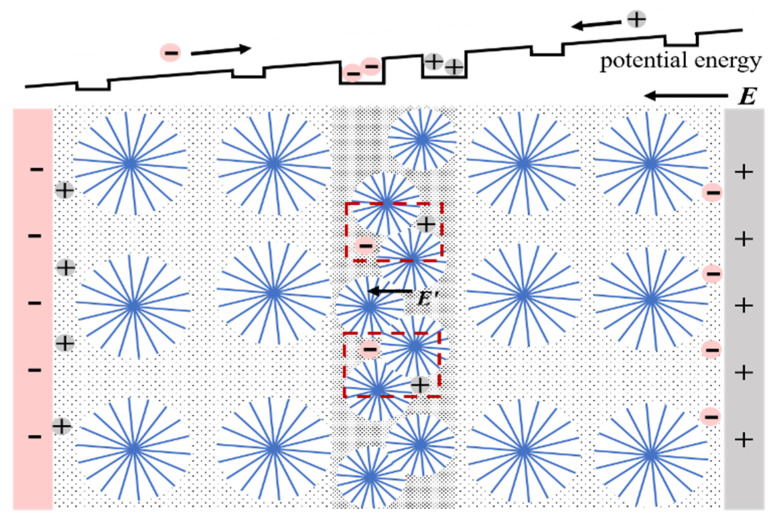
Schematic diagram of charge accumulation in H-XLPE bilayer.

Under an electric field *E*, the ionized charge near the electrode migrates directionally, which tends to occur in the amorphous region rather than the crystalline region. Nevertheless, the fused lamellas in the homo-junction region restrict the charge transport process caused by the thick lamella and high crystallinity, and the ends of macromolecular chains and vacancies can be considered as deep traps [29,30] that capture charge, as shown in Figure 2a. It could be the cause of charge accumulation in the H-XLPE bilayer. The local electric field *E*’ generated by the opposite polarity charge accumulated in the homo-junction region will increase and distort *E*, which reduces the DC dielectric strength of the H-XLPE bilayer, as is explained in Figure 10.

## 5. Conclusions

In this paper, the charge accumulation in the H-XLPE bilayer specimen is studied and the effects on electric field distribution and DC breakdown strength are discussed. Crystal morphology analysis, performed with the help of optics and thermal methods, is used to explain this phenomenon. The results show that the homo-junction region existing in the H-XLPE bilayer has two characteristics: a larger crystalline region that limits charge transport, and a higher number of deep traps that capture the charge. Both of these characteristics lead to the charge accumulation in H-XLPE bilayer. The accumulated charge generates a local electric field that distorts the original electric field; consequently, the DC breakdown strength of insulation material deteriorates. This work highlights the important inherent characteristics of factory joint return insulation that require further detailed investigation.

## Figures and Tables

**Table 1:** Microcrystal size of the various position of H-XLPE bilayer and XPLE (unit: nm).

	Specimen	H-XLPE			XLPE		
Lattice Orientation		1	2	3	1’	2’	3’
(110)		16.48	17.37	16.44	16.44	16.38	16.41
(200)		7.70	8.19	7.69	7.67	7.64	7.70

**Table 2:** Surface free energy of the various positions of H-XLPE bilayer and XPLE (unit: 10^−3^ J/m^2^).

	Specimen	H-XLPE			XLPE		
Lattice Orientation		1	2	3	1’	2’	3’
(110)		161.54	193.77	157.70	151.13	146.70	145.92
(200)		76.00	91.31	73.70	70.50	68.45	68.47

## Data Availability

The data presented in this study are available upon request from the corresponding author. The data are not publicly available due to fund requirements.
